# Drinking Water Quality and Public Health in the Kathmandu Valley, Nepal: Coliform Bacteria, Chemical Contaminants, and Health Status of Consumers

**DOI:** 10.1155/2022/3895859

**Published:** 2022-02-12

**Authors:** Bibudhendra Sarkar, Erika Mitchell, Seth Frisbie, Laurie Grigg, Sagar Adhikari, Rejina Maskey Byanju

**Affiliations:** ^1^Molecular Medicine, The Research Institute of The Hospital for Sick Children, University of Toronto, Toronto, Ontario, Canada; ^2^Department of Biochemistry, University of Toronto, Toronto, Ontario, Canada; ^3^Better Life Laboratories, Inc., Calais, VT, USA; ^4^Department of Chemistry and Biochemistry, Norwich University, Northfield, VT, USA; ^5^Department of Earth and Environmental Science, Norwich University, Northfield, VT, USA; ^6^International Centre for Integrated Mountain Development, Kathmandu, Nepal; ^7^Central Department of Environmental Science (Institute of Science and Technology), Tribhuvan University, Kathmandu, Nepal

## Abstract

Residents of Nepal's Kathmandu Valley draw drinking water from tube wells, dug wells, and stone spouts, all of which have been reported to have serious water quality issues. In this study, we analyzed drinking water samples from 35 tube wells, dug wells, stone spouts, and municipal tap water for bacterial and chemical contaminants, including total and fecal coliform, aluminum, arsenic, barium, beryllium, boron, cadmium, cobalt, chromium, copper, fluoride, iron, mercury, manganese, molybdenum, nickel, lead, antimony, selenium, thallium, uranium, vanadium, and zinc. We also asked a sampling of households who used these specific water sources to rate the taste of their water, list any waterborne diseases they were aware of, and share basic health information about household members. This survey provided us with information from 146 households and 603 individuals. We found widespread bacterial contamination of water sources, with 94% of sources having detectable total or fecal coliform. Nepal Drinking Water Quality Standards and World Health Organization (WHO) Drinking-Water Guidelines or health-based values were exceeded for aluminum (max = 0.53 mg/L), arsenic (max = 0.071 mg/L), iron (max = 7.22 mg/L), and manganese (max = 3.229 mg/L). The distribution of water sources with high arsenic, iron, and manganese appeared to be associated with floodplain deposits. Mixed effects logistic regression models were used to examine the interactions between social factors and water contaminants and their effects on household members' health. Consumers of water sources with both high and low concentrations of manganese were less likely to have a positive attitude towards school than those whose water sources had moderate concentrations of manganese. Social factors, especially education, played a large role in predicting individual health outcomes. Household taste ratings of drinking water were not correlated with iron or manganese concentrations, suggesting that WHO's reliance on aesthetic criteria for these contaminants instead of formal drinking-water guidelines may not be sufficient to protect public health.

## 1. Introduction

Accessing safe water is extremely difficult for the 1.7 million people of the Kathmandu Valley [1]. Although there is a municipal tap water system, even for those who have access to tap water in their homes, it is only available intermittently, especially after the 7.8-magnitude earthquake of April 2015. As a result, people gather and drink water from a variety of other sources, including dug wells, tube wells, and stone spouts (*dhunge dharas*) that access groundwater.

Unfortunately, water from these sources is frequently contaminated with bacteria, nitrate (NO_3_^−^), and metals at unsafe levels. A 2007 survey of Kathmandu drinking water sources detected unsafe levels of total coliform in 94% of all sources tested, and fecal coliform in 72%, as well as arsenic (As), mercury (Hg), manganese (Mn), and iron (Fe) in some sources at levels of concern for chronic exposures [2]. Unsafe concentrations of metals including arsenic, iron, manganese, and mercury have been reported in other studies [3–8]. Numerous studies have reported that bacterial contamination affected over 80% of samples, including water from dug wells, stone spouts, shallow tube wells, deep tube wells, tap water, and bottled water [6, 9–16].

The World Health Organization (WHO) notes that certain metals such as iron or manganese may impart off flavors, causing acceptability problems with consumers [17]. Water containing relatively high concentrations of calcium (Ca) and magnesium (Mg) is preferred in taste panel tests [18–20]. Laboratory studies have explored taste thresholds for various minerals such as aluminum, copper (Cu), iron, manganese, and zinc (Zn) [21–27]. However, less research has been done on the chemical content of water and consumer taste preferences outside the laboratory.

In their 2007 extensive study of water source types and contamination, Warner et al. found that bacterial contamination was most common in sources drawing water from the shallow aquifer, while deep groundwater sources tended to have less bacteria, but more iron and manganese, giving deep water sources a metallic taste [2]. They noted that Kathmandu consumers reported preferring to drink water from sources with lower iron concentrations, which meant greater exposures to bacterial contamination [2]. Studies in other regions have found no correlations between microbial contamination and consumer ratings of water taste [28–30]. In contrast, de Queiroz et al. found that familiarity of the water source characteristics (taste, odor, and color) plays a strong role in consumer preferences and risk perception, and Rupani et al. observed that a change in taste of the water was correlated with increased risk of gastrointestinal symptoms [31, 32]. In a study of household perceptions of water in Minnesota, Scher et al. found that higher manganese concentration was associated with greater concern about taste, odor, or color of the water, but that 54% of respondents whose well water had concentrations of Mn above 300 micrograms per liter (*μ*g/L) were not very concerned with the taste, odor, or color of their water [33].

Arsenic exposure through drinking water is known to cause a variety of diseases including cancers, hypertension, diabetes, neuropathy, and learning disabilities in children [34]. Lead exposure has also been associated with learning disabilities, hypertension, tooth loss, and diabetes [34]. Excessive manganese exposure through drinking water has been associated with learning disabilities in children and violent behaviors in adults [35–37]. Uranium (U) exposure through drinking water has been associated with increased blood pressure and bone turnover markers [38–40]. Chronic uranium exposure has also been associated with adverse neurodevelopmental effects [41, 42].

Waterborne diseases including typhoid, dysentery, and cholera are very common in the Kathmandu Valley [43]. Arsenicosis has been reported in other regions of the country where drinking water concentrations of arsenic are higher [44, 45]. In the Kathmandu Valley, although arsenic has been reported in drinking water at levels up to 0.265 milligrams per liter (mg/L) [46], cases of arsenicosis have not yet been reported. A number of studies have compared the presence of specific contaminants in drinking water to incidence of disease in consumers of that water [32, 47–52]. However, no studies from Kathmandu have reported connections between specific chemical contaminants in water and incidence of human disease.

In the present study, we analyzed samples from a broad selection of drinking water sources in the Kathmandu Valley and conducted a health and drinking water preference survey of the users of these sources. Our study goals were as follows:

1. Compare bacteria and metals concentrations and distributions to results from previous studies.
2. Investigate consumer preferences for taste—is taste helpful for choosing safe water?
3. Compare self-reported disease conditions to water contamination—are any specific contaminants associated with diseases or symptoms?

## 2. Materials and Methods

### 2.1. Study Region

**Figure 1:**
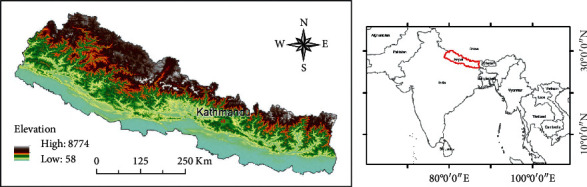
(a) Location and elevation map of Nepal and Kathmandu. (b) Location map of Asia and Nepal. Elevation data is from the SRTM 90 m digital elevation database [53].

The Kathmandu Valley is situated in central Nepal, a land-locked country bordering China in the north and India in the south, east, and west (see Figure 1(b)). The Kathmandu Valley includes 3 political districts: Kathmandu District, Lalitpur District, and Bhaktapur District. It is the political, economic, and cultural hub of Nepal, with a population of 9.7 million people [54]. The mean per capita income is US$2.61/day, compared to the countrywide mean of US$1.14/day [55]. For all of Nepal, the average years of schooling completed is 5.3 [56]; the literacy and schooling completion rate is higher in the Kathmandu Valley than in the remainder of the country [57].

The Kathmandu Valley stands at 1,425 meters (m) above sea level. It is surrounded by 4 mountain ranges: Shivapuri (2,732 m), Phulchowki (2,791 m), Nagarjun (2,732 m), and Chandragiri (2,551 m) [58]. The major river flowing through the valley is the Bagmati. Nepal is one of the most tectonically active regions in the world, as was evident during the devastating magnitude 7.8 Gorkha earthquake of April 25, 2015. Kathmandu is within a nappe along the Main Himalayan Thrust, which accommodates movement of the Indian Plate into the Asian Plate at a rate of 40–50 millimeters/year [59]. The Kathmandu Valley is an intermontane tectonic basin within the Lesser Himalayan Mountains; see Figure 1(a) [60]. The basin is filled with mostly Quaternary-aged (recent to Pleistocene in age) sediments up to 500 m thick, which overlie Precambrian and Paleozoic metamorphic rocks; see Figure 1(a) [61, 62]. The Pleistocene sediments in the valley are derived from a paleo-lake, which once filled the basin [60]. The lithology of these fluviolacustrine sediments has potential impacts on the propagation and degree of shaking of seismic waves [63], the reconstruction of past tectonic and climatic events [64], the susceptibility to groundwater pollution [62], and groundwater chemistry [65].

**Figure 2:**
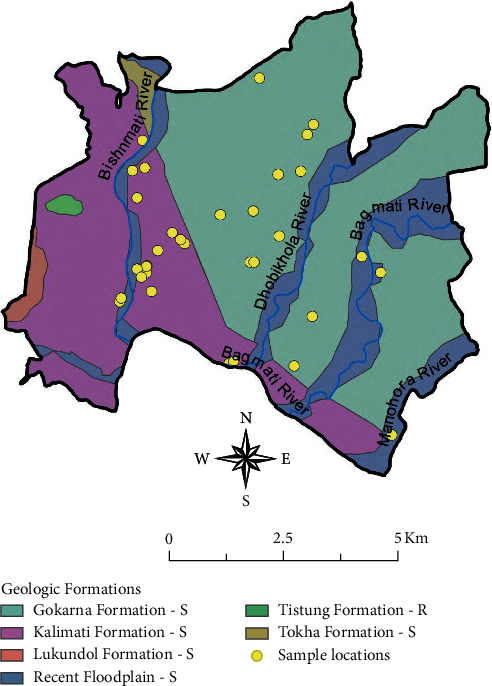
Location map of sample sites, major rivers, and geologic formations underlying the Kathmandu Valley. The mapping of geologic formations was based on the engineering and environmental geologic map by Shrestha et al. [61].

The northeastern portion of the study region is underlain by the 300 m-thick Gokarna Formation, which consists of permeable silty sands with some interbedded clay in the upper portion; see Figure 2 [61, 62]. This formation has been interpreted as deltaic, having formed during the lowering of the paleo-lake that once filled the basin [66]. The Gokarna Formation has been shown to be hydraulically connected with recent alluvium along the floodplains of the upper Bagmati and Manohara rivers [67]. The 450 m thick Kalimati Formation underlies much of the western portion of the study area. This formation is comprised of thick grey clay with beds of organic clay, fine sand, and peat and is lacustrine in origin [61, 62]. Recent floodplain deposits, which are found along the major rivers, contain unconsolidated clay, sand, and fine gravel [61, 62].

### 2.2. Water Sampling

Samples of drinking water were collected at 35 public drinking water sources (7 dug wells, 18 tube wells, 9 stone spouts, and 1 municipal tap) throughout the Kathmandu Valley during January-February of 2016 (see Figure 2); 1 sample was collected from each source. The dug wells are open water wells into which users lower a container into a shallow groundwater source to fill it. The tube wells are metal hand pumps that access shallow or deep groundwater through a vertical tube inserted into a narrow hole drilled into the ground. The stone spouts are public water fountains, often with ornately carved stone spouts, that provide access to groundwater with a continuous flow. The tap water sample was drawn from a metal tap connected to household plumbing accessing the municipal public water supply. All water samples were collected directly from these sources into sample bottles without further treatment in accordance with the regular water collection and usage practices of the consumers of these sources. That is, water samples were collected to represent ingestion exposure, which may include analytes from both the aquifer and the distribution system.

The number of samples was limited due to budgetary constraints, so the sample plan was carefully constructed to include samples throughout the Kathmandu Valley and from all major types of water sources. Our original sampling plan utilized stratified random sampling with sampling locations chosen a fixed distance from each other, but the immense destruction of roads and buildings caused by the April 2015 earthquake made access impossible at some locations. As a result, some of the random sample locations from a 2012-2013 field study [68] were also selected for resampling. The sampled sources were stratified so as to include the entire Kathmandu Valley; individual sources were selected randomly within this stratification.

At each water source, the sampling team recorded the Global Positioning System (GPS) coordinates and interviewed the source owner, caretaker, or a regular user to determine the age of the source, depth, and how many households use the source. The water was pumped or allowed to flow at full force for 5 minutes, and then samples were collected into two separate 250 milliliter (mL) sterilized glass sampling bottles, one for bacterial analyses and one for chemical analyses. The water samples were not filtered, because this was an exposure assessment of drinking water; our objective was to measure the concentrations of total chemicals, not just dissolved chemicals. The pH of the flowing water was tested with a pH meter (Milwaukee Instruments Martini pH55, Rocky Mount, NC, USA).

The sample bottles were stored in a cooler with ice and transported to the laboratory at Tribhuvan University in Kathmandu. Upon arrival at the university, the bacterial samples were analyzed immediately, and the metals samples were further preserved through the addition of concentrated nitric acid (HNO_3_) to a pH of less than 2. The chemical samples were then shipped to Vermont for chemical analysis at the Vermont Department of Health Laboratory (VDHL), a certified water testing laboratory.

### 2.3. Bacterial Analyses

Total coliform and fecal coliform contamination of samples were quantified with the membrane filtration method [17] at Tribhuvan University in Kathmandu. A membrane filter paper (0.45 micrometer porosity) was placed in a filter holder mounted on a funnel placed on a Buchner flask. The water sample was shaken, and then 100 mL of sample was poured into the funnel. The entire water sample was filtered through the paper under vacuum. A sterile absorbent pad was placed in a sterile Petri dish, and 2 mL of nutrient media (M-endo agar LES, HiMedia Laboratories, Pvt., Ltd., Mumbai, India) was added to the pad. After the water sample was filtered through the filter paper, the filter paper was transferred with sterile forceps and placed over the absorbent pad in the Petri dish. The dish was incubated in an inverted position at 37° Celsius for 24 hours. The filter paper was then removed and examined under a microscope, and the numbers of colonies were counted.

### 2.4. Chemical Analyses

The concentrations of all inorganic chemicals were measured at the VDHL in Colchester, Vermont. The VDHL is accredited by the National Environmental Laboratory Accreditation Program (NELAP). In addition, the VDHL is a certifying authority for drinking water quality testing under the Safe Drinking Water Act (SDWA) and Vermont (VT) Statute 18 V.S.A. § 501b [69].

The concentrations of aluminum, arsenic, barium (Ba), beryllium (Be), cadmium (Cd), cobalt (Co), chromium (Cr), copper, Mercury, manganese, molybdenum (Mo), nickel (Ni), lead (Pb), antimony (Sb), selenium (Se), thallium (Tl), uranium, vanadium (V), and zinc were measured by Inductively Coupled Plasma/Mass Spectrometry (ICP/MS) using United States Environmental Protection Agency (U.S. EPA) Method 200.8 [70]. The ICP/MS was a PerkinElmer, Inc. ELAN DRC II with an Elemental Scientific Inc. (ESI) SC-4DX FAST sample injection system (PerkinElmer ELAN DRC II, Waltham, MA, USA).

The concentrations of boron (B) were also measured by this ICP/MS. However, boron is not listed as a U.S. EPA Method 200.8 analyte [70], most likely because of its memory effect [71, 72]. That is, boron from one sample can carry over to the next sample with some laboratory methods. For our analyses, the memory effect was controlled by rinsing the sample introduction system with a mixture of 2% HNO_3_ and 1 mg/L of gold (Au). The ^10^B and ^11^B isotopes were measured against a ^6^Li (lithium) internal standard. Otherwise, the calibration and quality control were the same as those used by U.S. EPA Method 200.8.

The concentrations of iron were measured by flame atomic absorption spectrometry (FAAS) using VDHL procedure P-EC-402 Rev. 6, an adaptation of Standard Methods for the Examination of Water and Wastewater method 3111B [73]. A PerkinElmer Model AAnalyst 400 Atomic Absorption Spectrophotometer (AAS) was used for these analyses (PerkinElmer Model AAnalyst AAS, Waltham, MA, USA).

The concentrations of fluoride anion (F^−^) were measured by ion selective electrode using VDHL procedure P-EC-4500-F-G Rev. 5, an adaptation of Lachat method 10-109-12-2-A [74]. A Lachat Flow Injection Analyzer (FIA) QuikChem 8500 Series 2 was used for these analyses (Lachat FIA QuikChem 8500 Series 2, Loveland, CO, USA).

The quality assurance/quality control (QA/QC) procedures for this project included the analysis of known additions of standard to samples, and reagent blanks to monitor accuracy and the analysis of duplicate samples to monitor precision.

### 2.5. Household Surveys

At each of the first 30 water sources where samples were collected, 5 consecutive users of the source were asked about their household water use. Using a structured interview, a trained Tribhuvan University student volunteer asked respondents how frequently the household used the source for drinking, their awareness of waterborne diseases, their opinion of the water taste, and their reasons for choosing between this source and another source. For example, regarding taste, users were asked, “What do you think of the taste of the water from this other source? (good/OK/bad).” Users were also asked about their household food security, house construction material, and the age, health status, tobacco use, number of years of schooling completed, and attitude towards schooling for each household member. The interviews were conducted in Nepali, and answers were recorded in English; the survey questions are provided in Supplementary Material 1.

Before each survey began, each respondent was informed about the nature of the study, that participation in the study was voluntary, that no identifying information would be used in study reports, and that he or she could decline to answer any questions. Respondents gave verbal consent before the interviews proceeded; the informed consent script is provided in Supplementary Material 2. Respondents were provided with analyses of the water from the sources that they used as compensation for participating in the study. In all, 146 household surveys were completed, including health and education data for 603 individuals. The research team conducted their work in compliance with all scientific norms and standard of ethics as required and maintained by the Institute of Science and Technology, Tribhuvan University, Nepal.

### 2.6. Statistical Analyses

Statistical analyses were performed using R version 4.0.4 “Lost Library Book,” released on February 15, 2021. Distributions of individual numerical variables were tested for normality before statistical tests were performed. Parametric tests were used for variables with normal distributions, and nonparametric tests were used for variables with nonnormal distributions. However, Welch's *t*-tests were used for comparing means amongst 2 groups containing at least 15 members each, regardless of the normality test result for the variable [75]. For Kruskal-Wallis multiple comparison tests, *p* values were adjusted by the Holm method. Significance tests for other multiple comparisons within groups of data were corrected for multiple comparisons using Benjamini and Hochberg's False Detection Rate (FDR) method assuming *α* = 0.5 [76]. Significant results after corrections for multiple comparisons are highlighted in bold in the text.

For each water source, a numerical taste rating was constructed by converting the household subjective categorical scores for taste into consecutive numbers (1–3) and averaging all the numerical scores for the source [77]. Possible effects and interactions of water characteristics and household characteristics on health conditions (e.g., hypertension) were examined through mixed effects logistic regression models in R. Due to the hierarchical method of sampling, both household membership and water source were treated as random variables for the logistic regression models. However, water source was not found to have significant effects in any of these models, so only household membership was retained as a fixed effect.

For analyses of economic effects on health, an asset-based measure of relative household socio-economic status (SES) was constructed by combining house material, whether at least one person in the household had completed 5 years of schooling, and perceived household food security as reported by the subjects [78, 79]. That is, households with no food insecurity, who lived in concrete buildings, and had at least one member with 5 years or more of education were classified as “Higher” SES for the purposes of this study, and all others were classified as “Lower” SES. These are relative terms specific to this study and likely do not correspond to specific measures of wealth.

## 3. Results and Discussion

### 3.1. Water Sources

Of the 35 water sources sampled, 12 were privately owned, and 23 were publicly owned. The private wells had been constructed by individual owners at their own expense for the benefit of themselves, family members, and guests; individual owners controlled access to these wells. The publicly owned wells had been constructed by the government or charity organizations and were available for all to use. On average, publicly owned wells had been in use longer (*n* = 23, Median = 100 years) than privately owned wells (*n* = 12, Median = 15.5 years; *N* = 35, *W* = 64, **p=0.010**) and served more households (*n* = 23, Median = 90) than privately owned wells (*n* = 23, Median = 6.5; *N* = 35, *W* = 15, **p<0.001**); see Table 1. Tap water was not available to many residents, so only 1 sample was collected from this source; this single tap water sample may not be representative of all tap water in Kathmandu Valley but serves as an example of the possible tap water quality that may be obtained in this region.

### 3.2. Bacterial Contaminants

**Figure 3:**
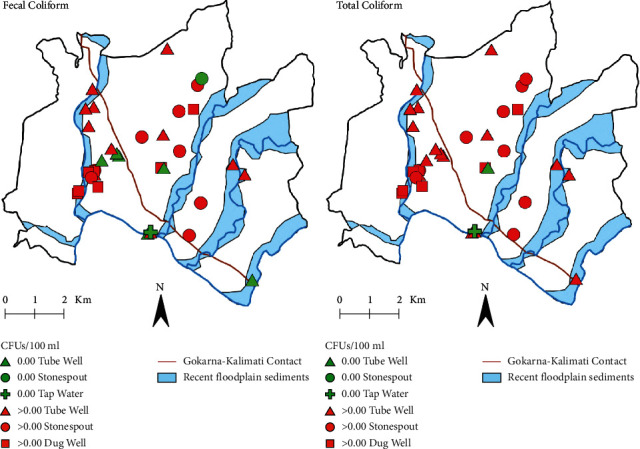
Concentration maps of total and fecal coliform for each sample site. Map symbols indicate the type of water source as well as concentration value. The geologic contact and extent of floodplain sediments are from Shrestha et al. [61].

Bacterial contamination was widespread, with 94% of the sources (33/35) having positive results for total coliform, fecal coliform, or both (see Table 1 and Figure 3). Total coliform counts ranged from 0 to “too many to count,” with a mean count of 600 colony forming units (CFUs)/100 mL, and a maximum count of 1,700 CFUs/100 mL; only 2 sources had a total coliform count of 0 CFUs/100 mL; see Table 1. For the purposes of statistical analyses, samples rated “too many to count” were given the nominal value of the maximum counted number, 1,700 CFUs/100 mL. Fecal coliform counts ranged from 0 to “too many to count,” with a mean count of 378 CFUs/100 mL, and a maximum count of 3,025 CFUs/100 mL; 9 sources (26%) had a fecal coliform count of 0; see Table 1. For the purposes of statistical analyses, samples rated “too many to count” were given the nominal value of the maximum counted number, 3,025 CFUs/100 mL.

There were no significant differences between the type of ownership of the water sources (public, private) and total (*N* = 35, *W* = 114.5, *p* = 0.421) or fecal (*N* = 35, *W* = 141, *p* = 0.930) coliform counts. Kruskal–Wallis Tests found no significant differences between the type of water source (tubewell, dugwell, stone spout) and total (*χ*^2^(2,33) = 2.05, *p* = 0.359) or fecal (*χ*^2^(2,33) = 4.11, *p* = 0.128) coliform counts. No correlation was found between the number of households served by a water source and total (*r*_*s*_(33) = 0.08, *p* = 0.637) or fecal (*r*_*s*_(33) = −0.18, *p* = 0.301) coliform counts. Depth of the water source was not correlated with total (*r*_*s*_(33) = −0.37, *p* = 0.073) or fecal coliform counts (*r*_*s*_(33) = −0.11, *p* = 0.603). In a 2014 Nepal-wide drinking water survey, Kandel et al. found bacterial contamination in over 80% of all sources, with no significant differences in contamination between “improved” water sources compared to “unimproved sources” [9]. In the Kandel et al. study, “improved” sources included piped water, tube wells, protected dug wells, protected springs, and rain water, while “unimproved” sources included unprotected dug wells, tanker trucks, surface waters, and bottled water [9]. However, Warner et al. found that water from stone spouts and dug wells in the Kathmandu Valley had more bacterial contamination than water from deep tube wells and noted that sanitation and waste management in the region are virtually nonexistent [2]. Lack of significant correlation between source type and bacterial contamination in the present study may have been due to the limited number of samples from the individual source types.

### 3.3. Chemical Contaminants

**Figure 4:**
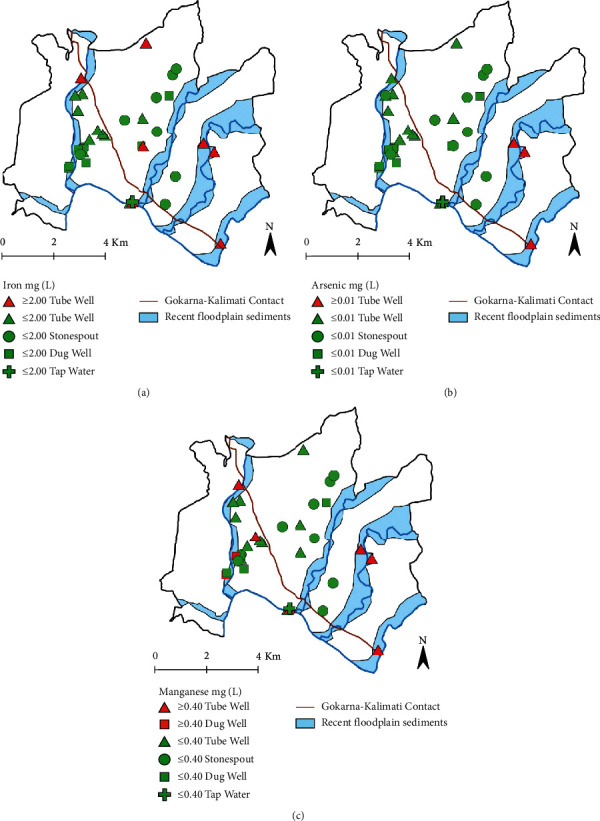
Concentration maps of iron, arsenic, and manganese for each sample site. Map symbols indicate the type of water source as well as concentration value. Green symbols indicate values that fall below WHO health-based values (HBVs); red symbols indicate sites that are above WHO HBVs. The geologic contact and extent of floodplain sediments is from Shrestha et al. [61]. (a) Iron. (b) Arsenic. (c) Manganese.

Concentrations of elemental chemical contaminants from sampled sources, Nepal Drinking Water Quality Standards (DWQS), WHO health-based values (HBV), and WHO drinking-water guidelines (DWG) are summarized in Table 2 and Figure 4. Nepal DWQSs were exceeded for aluminum, arsenic, iron, and manganese. WHO HBVs for chemical contaminants were exceeded for arsenic, manganese, and iron, while WHO DWGs were exceeded only for arsenic. Concentrations of antimony, barium, beryllium, boron, cadmium, chromium, cobalt, copper, fluoride, lead, mercury, molybdenum, nickel, selenium, thallium, uranium, and zinc were within Nepal DWQSs, WHO HBVs, and WHO DWGs for all samples.

The WHO DWG of 0.01 mg/L for arsenic is based on treatment performance and analytical achievability rather than health effects [17, 81]. The HBV for arsenic concentration in drinking water is much lower, 0.00017 mg/L [84, 85] but the WHO deems this HBV not achievable due to analytical and treatment performance constraints. Of the 35 samples tested, at least 28 (80%) exceeded this HBV, while 3 (9%) exceeded the WHO DWG, and 1 (3%) exceeded the Nepal DWQS for arsenic. A Kruskal–Wallis test found no significant differences in arsenic concentration between tube wells, dug wells, or stone spouts (*χ*^2^(2,33) = 2.81, *p* = 0.245) or between public and privately owned wells (*N* = 35, *W* = 94, *p* = 0.132). The concentration of arsenic was not significantly correlated with the depth of the water source (*r*_*s*_(33) = 0.27, *p* = 0.197) or the number of years the source has been in use (*r*_*s*_(33) = 0.14, *p* = 0.427). The concentration of arsenic was significantly positively correlated with boron (*r*_*s*_(33) = 0.47, **p** **=** **0.004**) and thallium (*r*_*s*_(33) = 0.49, **p** **=** **0.003**) concentrations. Previous surveys in Kathmandu have found higher concentrations of arsenic in deeper tube wells than shallow tube wells [2, 8]. Previous studies have also reported boron, and thallium codeposited with arsenic in the Kathmandu Valley [86, 87].

The WHO withdrew its 0.4 mg/L drinking water guideline for manganese in 2011 on the grounds that the health-based value of 0.4 mg/L “is well above concentrations of manganese normally found in drinking-water” [17]. Subsequent surveys showed that manganese concentrations in drinking water often exceed 0.4 mg/L [35, 83]. Of the 35 samples tested, 34% exceeded the Nepal DWQS of 0.2 mg/L for manganese, and 20% exceeded the WHO HBV of 0.4 mg/L for manganese. In our samples, the concentration of manganese varied according to the water source type (tube wells, dug wells, stone spouts; *χ*^2^(1,32), = 11.13, **p** **=** **0.004**); Dunn post hoc tests showed that water from tube wells (Median = 0.237 mg/L) contained more manganese than water from stone spouts (Median = 0.028 mg/L; *Z*(26) = −3.25, **p** **=** **0.003**). There was no difference in manganese concentration between public and privately owned water sources (*N* = 35, *W* = 192, *p* = 0.062). Manganese concentrations were not correlated with depth of the water source (*r*_*s*_(33) = 0.14, *p* = 0.519). Manganese concentrations were positively correlated with cadmium (*r*_*s*_(33) = 0.44, **p** **=** **0.008**), cobalt (*r*_*s*_(33) = 0.50, **p** = 0**.002**), iron (*r*_*s*_(33) = 0.74, **p** **<** **0.001**), and mercury (*r*_*s*_(33) = 0.50, **p** **=** **0.003**) concentrations, and negatively correlated with the number of years the source has been in use (*r*(33) = −0.44, **p** **=** **0.008**).

The WHO does not have a DWG for iron but notes that an HBV of 2 mg/L “does not present a hazard to health” [17, 81]. Of the 35 samples tested, 20% exceeded this HBV of 2 mg/L, while 46% exceeded the Nepal DWQS for iron of 0.3 mg/L. In our samples, the concentration of iron varied according to the water source type (tube wells, dug wells, stone spouts; *χ*^2^(1,32), = 15.92, **p** **<** **0.001**); Dunn post hoc tests showed that water from tube wells (Median = 0.61 mg/L) contained more iron than water from dug wells (Median = 0.15 mg/L; *Z*(24) = −2.54, **p** **=** **0.022**) or stone spouts (Median = 0.05 mg/L; *Z*(26) = −3.70, **p** **=** **0.001**). Iron concentration was not associated with the ownership type of the water sources (public/private) (*N* = 35, *W* = 186.5, *p* = 0.092). Iron concentrations were positively correlated with barium (*r*_*s*_(33) = 0.52, **p** **=** **0.001**), and mercury (*r*_*s*_(33) = 0.45, **p** **=** **0.007**), and negatively correlated with the age of the source (*r*_*s*_(33) = −0.53, **p** **=** **0.001**) and vanadium (*r*_*s*_(33) = −0.45, **p** **=** **0.006**).

### 3.4. Spatial Distribution of Chemical Results

Of the 7 samples with unsafe iron concentrations (>2.0 mg/L), 5 were located near a major river within recent floodplain sediments, and 2 were within the deltaic sediments of the Gokarna Formation (see Figure 4). With the exception of 1 sample, all the samples from the floodplain sediments had concentrations between 5.01 and 8.00 mg/L, while the samples from the deltaic sediments fell between the range of 2.01 and 5.00 mg/L.

The 3 samples showing unsafe arsenic concentrations (>0.01 mg/L) were all located in the eastern portion of the study region within recent floodplain deposits (see Figure 4). Two of the samples were located within 0.5 kilometers of each other along the Upper Bagmati River. The third sample was located along the Manohara River.

The distribution of samples containing unsafe concentrations of manganese (>0.4 mg/L) initially appeared to have less spatial consistency than iron or arsenic because they are found east and west of the major geologic contact in the region (see Figure 4). However, upon closer examination, it is apparent that, of the 8 samples with high concentrations of manganese, all but 1 are from recent floodplain sediments and have a concentration between 0.41 and 2.0 mg/L. The 1 sample not found within a floodplain is within the lacustrine-derived Kalimati Formation and has a slightly higher concentration (3.23 mg/L).

The higher concentrations of iron, manganese, and arsenic in groundwater in contact with floodplain sediments further support the hypothesis that these metals are being released under reducing conditions in these aquifers [65, 88]. These sediments contain organic matter [65]; heterotrophic microorganisms feeding on this organic matter consume the dissolved oxygen, leading to reducing conditions. Under reducing conditions, insoluble As(V), Fe(III), and Mn(IV) are reduced to soluble As(III), Fe(II), and Mn(II). That is, these reducing conditions cause the arsenic, iron, and manganese in the geologic material to dissolve into the water, elevating the concentrations of these metals in water sources in contact with floodplain sediments of this valley. The presence of elevated iron from 2 sites within the Gokarna Formation may be explained by the high permeability of this formation and its hydraulic connectivity with floodplain sediments [67], which could serve as source for groundwater enriched in soluble iron.

### 3.5. Taste

Water source type (tube well, dug well, and stone spout) did not affect taste ratings averaged across the responding households for each water source (*F*(2, 33) = 0.79, *p* = 0.463) or ownership type (public, private) of the water source (*t*(34) = −1.40, *p* = 0.177). Average taste ratings for the water sources were not significantly correlated with depth (*r*_*s*_(33) = 0.26, *p* = 0.221), pH (*r*_*s*_(33) = −0.08, *p* = 0.631), temperature (*r*_*s*_(33) = −0.24, *p* = 0.166), or age of the source (*r*_*s*_(33) = 0.10, *p* = 0.580). Similarly, total (*r*_*s*_(33) = 0.13, *p* = 0.470) and fecal (*r*_*s*_(33) = −0.26, *p* = 0.130) coliform counts did not affect taste scores, nor did concentrations of aluminum (*r*_*s*_(33) = −0.10, *p* = 0.550), arsenic (*r*_*s*_(33) = 0.12, *p* = 0.478), copper (*r*_*s*_(33) = 0.32, *p* = 0.060), iron (*r*_*s*_(33) = −0.19, *p* = 0.284), manganese (*r*_*s*_(33) = −0.06, *p* = 0.753), zinc (*r*_*s*_(33) = −0.04, *p* = 0.803), or any other chemicals that we tested for (data not shown).

Lack of an effect of water source type on taste is in contrast to Pattanayak et al. [89], who reported that Kathmandu Valley consumers gave positive taste ratings to stone spout water and private water sources, and negative ratings to tap water, and Warner et al. [2], who noted that consumers frequently reported that water from shallow tube wells and dug wells had a bad taste. However, the Pattanayak et al. and Warner et al. studies did not mention statistical tests for significance for their reported associations between source type and taste [2, 89].

In the 2017 addendum to the 2011 WHO DWGs, WHO revised their 2011 reason for not promulgating a DWG for manganese, stating “As this health-based value is well above concentrations of manganese normally causing acceptability problems in drinking-water…, it is not considered necessary to derive a formal guideline value” [17, 81]. While the WHO has set an HBV for Mn at 0.4 mg/L, it states that Mn at concentrations of 0.1 mg/L imparts an undesirable taste to beverages [17]. Sain and Dietrich reported a 50% Best Estimate Taste threshold for Mn(II) of 165 mg/L, orders of magnitude higher than the WHO's stated taste threshold for total Mn, but noted that taste thresholds may be dependent on subjects, methods, and conditions [25]. Of the water samples that we tested, none contained more than 165 mg of Mn/L, and manganese concentration was not associated with taste ratings of the samples. The maximum concentration of Mn in our study was 3.229 mg/L (Table 2). A survey of Mn in well water in Minnesota found that household concern with the taste of their water was correlated with Mn concentration, but that 54% of households with water above Mn above 300 *μ*g/L were not very concerned about the taste, odor, or color of their water [33].

Regarding iron, WHO states “The taste and appearance of drinking-water will usually be affected below [2 mg/L]” [17, 81]. Cohen et al. (1960) reported that 50% of their panel detected the taste of iron at 8.8 ppm [8.8 mg of iron/L], considerably higher than the 2 mg/L concentration mentioned by the WHO [21]. None of our samples exceeded 8.8 mg of iron/L, and iron concentration was not associated with taste ratings of the samples.

Lack of correlation between manganese concentration and overall water taste scores or iron concentration and overall water taste scores in the present study draws into question the assumption by the WHO that taste acceptability may be sufficient to deter consumers from using water sources with unhealthy concentrations of these metals [17, 81]. Whelton et al. examined many factors, including Total Dissolved Solids (TDS), temperature, and consistency that may affect drinking water taste ratings [90]. It is possible that the presence or absence of minerals in the water such as sodium (Na) or magnesium may overwhelm taste effects due to Mn or Fe. Although it is likely that the oxidation state of Mn and Fe affect taste ratings and toxicity, WHO HBVs and aesthetic criteria for these metals refer only to the total concentrations, not the concentrations of various Mn and Fe ions. Thus, we examined total concentrations of metal contaminants and did not do speciation studies to determine oxidation states.

### 3.6. Households

Average household size in this study was 4.2 people, and average years of schooling for people over 15 years old was 8.9 years. According to the 2011 National Population and Housing Census, average household size for Kathmandu Valley was 3.71 in 2011 [91], while average years of schooling for people over 15 years old nationwide was 5.3 [56]. Of the 146 households using the sampled water sources, 39 (27%) were “Lower” SES and 107 (73%) “Higher” in our classification scheme, which gave equal weight to food security, housing material, and whether at least one household member had completed 5 years of schooling. The proportion of households that have Higher SES rather than Lower SES did not differ by the type (tube well, dug well, and stone spout) (*χ*^2^(2,145) = 0.62, *p* = 0.624) or ownership of the water source (*χ*^2^(1,145) = 2.62, *p* = 0.106). The difference between the number of households served by water sources used by Higher SES households did not differ from those used by Lower SES households (*N* = 146, *W* = 2,691, *p* = 0.007) after correction for FDR, nor did total (*N* = 146, *W* = 1,639, *p* = 0.048) or fecal coliform counts (*N* = 146, *W* = 1,750, *p* = 0.133).

When asked if they were aware of any disease caused by water, 73% of household respondents (106/146) said “yes.” Those answering “yes” (106) were asked to name some waterborne diseases. All respondents answering “yes” mentioned at least 1 scientifically recognized waterborne disease; the most commonly named waterborne disease was diarrhea (73 respondents), followed by dysentery, typhoid, and cholera (23, 23, and 22 respondents, respectively). Awareness of waterborne disease was associated with Higher SES (*χ*^2^(1,145) = 10.74, **p** **=** **0.001**). The most educated household member of respondents who were aware of waterborne diseases had significantly more years of schooling (*n* = 106, *M* = 12.9, SD = 3.92) than the most educated members of households of respondents who were not aware of waterborne diseases (*n* = 40, *M* = 9.9, SD = 4.69; *t*(145) = −3.55, **p** **=** **0.001**). There was no difference in total (*t*(145) = −0.07, *p* = 0.946) or fecal (*t*(145) = 1.61, *p* = 0.113) coliform counts in water used by respondents who were aware of waterborne diseases or not aware. In contrast, 90% of schoolchildren in Dolakha and Ramechhap districts of Nepal were reported to be aware of waterborne disease [92] as were 100% of “jar water” (commercially supplied 20 L refillable bottles) users in Kathmandu Valley [15].

Interestingly, 27/146 respondents in our survey (18%) mentioned common cold virus as a waterborne disease; this was the only common irrelevant answer to the question about diseases caused by water, and it was the second-most mentioned disease after diarrhea. Based on this widespread misconception of the cold virus as a waterborne disease, we further analyzed the health reports to confirm that there was no actual basis for associating the common cold virus with any characteristics of the water sources. There was no difference between years of education completed by the most educated household member of respondents who mentioned irrelevant diseases when asked to name waterborne diseases (*t*(145) = −1.86, *p* = 0.065). Associating drinking water with nonrelevant diseases such as common cold virus was also reported as extremely common in Tamil Nadu, India, where it was suggested that the concept of “hot” and “cold” in local folk medicine may be a factor [93].

The potential for second-hand smoke exposure was present in 39/135 (29%) of households, those that included at least 1 tobacco smoker and had at least 2 household members. The proportion of households with second-hand smoke exposure potential did not differ between Lower SES and Higher SES households (*χ*^2^(1,134) = 0.18, *p* = 0.669). There was no difference in years of education held by the most educated household member in households with or without second-hand smoke exposure potential (*t*(134) = 1.26, *p* = 0.212). The second-hand smoke exposure prevalence in this study is comparable to other surveys of tobacco use in Nepal (36.1%: [94]).

When asked about other drinking water sources, 91% of the households (131/146) reported using other water sources at least some of the time. The other water sources included bottled water (mentioned by 60% of households), tanker truck water (16%), and tap water (10%). Of the 133 households that had access to alternative water sources, 62% expressed a preference for the source that was sampled, 14% expressed a preference for bottled water, 12% for tanker water, and 8% for tap water.

When asked to explain the reasons for their preference between water sources, 47% of respondents mentioned taste, 34% mentioned cost, 24% access or availability, 2% distance, and 2% quality (some respondents provided more than one reason). A greater proportion of Lower SES households than Higher SES households mentioned cost (*χ*^2^(1,145) = 6.45, **p** **=** **0.011**) and taste (*χ*^2^(1,145) = 9.77, **p** **=** **0.002**) as factors in their choice of water sources. There was no difference in years of education completed by the most educated household member amongst households who mentioned either cost (*t*(145) = 1.57, *p* = 0.120) or taste (*t*(145) = 1.64, *p* = 0.103).

As reasons for water source preferences, the most educated person in households who rated their water as having a “bad” taste had completed more years of education (*n* = 15, Median = 15) than the most educated person in households who rated their water as having an “OK” (*n* = 43, Median = 12) or “good” (*n* = 88, Median = 12) taste (*χ*^2^(2,145) = 8.58, **p** **=** **0.014**).

### 3.7. Individuals

Health status and education characteristics of subjects are summarized in Table S1. Of the 591 individuals included in the household surveys with gender specified, 51% were male. For the 493 subjects aged 15 years and older, the average years of schooling was 8.9 years (SD = 5.9 years). For the 591 subjects of all ages whose gender was specified, males had completed more years of schooling (*n* = 299, *M* = 8.6, SD = 5.8) than females (*n* = 292, *M* = 7.2, SD = 5.9; *t*(590) = −2.88, **p** **=** **0.004**).

Because smoking and exposure to second-hand smoke could be associated with numerous health effects and were potential confounders for effects due to water contaminants, the health surveys examined reported tobacco use by individuals and within households. According to the surveys, 8% of the household members were smokers and 2% used betel; the youngest reported tobacco user was 12 years old. Smoking prevalence was similar in urban areas of Nepal at 12% [94]. Of the 542 subjects who did not use tobacco, 110 (20%) lived in households that included smokers and, hence, had the potential for household second-hand smoke exposure.

Factors associated with reported tobacco use were explored further to facilitate subsequent study of possible interactions between tobacco use, second-hand smoke exposure, gender, age, schooling, and water contaminants on specific health conditions. Of the 516 subjects who were 12 years and older and whose gender was specified, a greater proportion of males were reported to use tobacco than females (*χ*^2^(1,515) = 36.38, **p** **<** **0.001**). Among subjects 12 years and older, the mean age of reported tobacco users (*n* = 61, *M* = 43.8 years, SD = 14.0) was higher than the mean age of reported non-users (*n* = 465, *M* = 33.3 years, SD = 15.6; *t*(525) = −5.44, **p** **<** **0.001**). Among subjects 12 years and older, the mean years of education was lower for tobacco users (*n* = 61, *M* = 7.3 years, SD = 5.8) than for non-users (*n* = 465, *M* = 9.0 years, SD = 5.8; (*t*(525) = 2.09, **p** **=** **0.036**).

Mixed effects logistic regression models were examined to explore possible associations between gender, age, education, and tobacco use. Male gender and age were found to have significant effects on the odds ratio (OR) of using tobacco after controlling for household membership; see Table S2. Associations between tobacco use and male gender, older age, and lower SES have been found in other tobacco use surveys in Nepal [94–97].

Of the 603 subjects, 337 (56%) were reported as having disease symptoms or chronic diseases at the time of the survey, with 0–4 specific diseases named for each affected individual. The most commonly reported diseases or symptoms were common cold (101 = 17%), gastrointestinal (GI) symptoms within the previous 4 weeks (105 = 17%, including diarrhea, nausea, vomiting, or stomachache), headache (48 = 8%), and hypertension (31 = 5%). It must be stressed that these figures represent reports by a single household member and were not confirmed by medical examinations by our team members. In particular, the reported prevalence ratio of hypertension most likely represents only the proportion of the population who had been previously diagnosed through medical care and chose to report this to our survey team. Since hypertension often does not cause noticeable symptoms, people who are not under regular medical supervision may not be aware that they have hypertension and were not identified in our survey.

### 3.8. Health Status Analyses

Based on the household survey results, we selected GI symptoms, reported hypertension, common cold, and schooling attitude for further analyses to see which of these might be associated with specific contaminants in the drinking water. We selected GI symptoms, hypertension, and common cold because these conditions were reported commonly enough to have the potential for sufficient power in our population for statistical tests, and each may also have either a causal or perceived link with a drinking water contaminant. Chronic exposures to arsenic [98] or uranium [38, 40] in drinking water have been linked to increased blood pressure. In the case of common cold, there is no medically known reason why this disease would be linked to drinking water, but subjects expressed a strong belief that it is a waterborne disease (it was the second-most named disease after diarrhea to be identified as waterborne by survey respondents); thus, we felt it prudent to check for any possible associations between common cold and water sources or contaminants.

Schooling attitude was used as a proxy measure indicating the potential for learning disabilities and behavioral problems, such as Attention Deficit Hyperactivity Disorder (ADHD) that might interfere with schooling. Students with learning disabilities or behavioral problems have been reported to have negative attitudes towards schooling [99, 100]. While schooling attitude is not a direct or specific indicator of learning disabilities or behavioral problems, this measure does not require extensive psychological testing or teacher interviews, and it can be used with people of all ages, not only students currently enrolled in school. Chronic arsenic and manganese exposures in drinking water have been associated with learning disabilities, behavioral problems, and IQ deficits in children [101–110]. Chronic uranium exposure may also be associated with adverse neurodevelopmental effects [41, 42].

#### 3.8.1. GI Symptoms

The overall prevalence of reported GI symptoms within the previous 4 weeks was 17% in the study population; this was within the range of prevalence reported in other Kathmandu Valley surveys 7.8% (diarrhea) to 57% [111, 112]. Reported GI symptom prevalence was 23% for those who regularly used stone spouts, 18% for those who used dug wells, and 14% for those who used tube wells; this difference was not significant after correcting for FDR (*χ*^2^(2,602) = 6.89, *p* = 0.032). There was no difference in the number of households served by water sources used by subjects reporting GI symptoms and those not reporting GI symptoms (*t*(602) = −1.48, *p* = 0.140). There was no difference between total (*t*(602) = 0.43, *p* = 0.670) and fecal (*t*(602) = 0.54, *p* = 0.589) coliform counts in water used by subjects who reported having GI symptoms and those who did not. Similarly, there were no differences in concentrations of antimony, arsenic, boron, barium, copper, iron, lead, manganese, uranium, vanadium, or zinc in water used by subjects who reported having GI symptoms and those who did not (data not shown).

The fact that we did not find a significant association between drinking water coliform counts (total or fecal) and reported GI symptoms was not entirely unexpected, since similar studies in India and Mexico also did not find direct associations between water bacterial indicators and GI symptoms [32, 47]. A study in Alabama found that drinking water bacterial indicators were associated specifically with vomiting and diarrhea prevalence, but not with general GI symptoms [30]. In contrast, some studies have found associations between diarrhea and *E. coli* counts in drinking water [113, 114]. Levy et al. note that because diarrhea is a general symptom with many causes, and drinking water represents only one type of exposure to pathogenic organisms, associations between drinking water pathogens and disease incidence may be weak and difficult to detect unless sample sizes are sufficiently large [113].

Subjects from Lower SES households were no more likely to report GI symptoms over the last 4 weeks than subjects from Higher SES households after correcting for FDR (*χ*^2^(1,602) = 3.95, *p* = 0.047), and there was no difference in household size between subjects who reported GI symptoms and those who did not report (*t*(602) = 2.05, *p* = 0.042), after correcting for FDR. The most educated household member of subjects who reported GI symptoms had completed fewer years of education (*n* = 105, *M* = 11.3 years, SD = 4.2 years) than the most educated household member of subjects who did not report GI symptoms (*n* = 498, *M* = 12.7, SD = 4.1 years; *t*(602) = 3.10, **p** **=** **0.002**). There were no significant differences in GI symptom prevalence among households who rated the taste of their primary water source as “poor,” “OK,” or “good” (*χ*^2^(2,145) = 0.69, *p* = 0.710). For subjects aged 15 years and older, there was no difference in average years of schooling for those who reported GI symptoms within the previous 4 weeks than those who did not, after correcting for FDR (*t*(492) = 2.19, *p* = 0.031). An examination of mixed effects logistic regression models did not find any significant noncollinear predictors for prevalence of GI symptoms from the predictors listed above (data not shown). Lower SES and less education have been associated with increased diarrhea prevalence in Kathmandu Valley [111, 115], while Rai et al. (2004) found that prevalence of gastroenteritis was greater in children from larger households [116].

#### 3.8.2. Hypertension

Prevalence of reported hypertension was 5% across all subjects. Since this was reported, not measured hypertension, and included both first-hand (interview subjects) and second-hand (household members of interview subjects) reports, reported prevalence was most likely lower than actual prevalence. In studies in which blood pressure was measured in adults, higher prevalence of hypertension has been reported (29% for urban Nepal: [94]; 32.5% for Kathmandu Valley: [117]).

Reported hypertension prevalence was 13% for those who used dug wells, 4% for those who used stone spouts, and 4% for those who used tube wells. The association between water source type and subjects reporting hypertension was significant (*χ*^2^(2,602) = 9.71, **p** **=** **0.008**). Total coliform counts in a subject's water source did not differ between subjects who reported hypertension and those who did not (*t*(602) = −0.34, *p* = 0.734), but fecal coliform counts were lower in the water of subjects who reported hypertension (*n* = 31, *M* = 105.6, SD = 244.8) than those who did not (*n* = 572, *M* = 359.9, SD = 867.3; *t*(602) = 4.46, **p** **<** **0.001**). The concentrations of As (*n* = 31, *M* = 0.005 mg/L, SD = 0.007), Mn (*n* = 31, *M* = 0.401 mg/L, SD = 0.728 mg/L), and U (*n* = 31, *M* = 0.0007 mg/L, SD = 0.0006 mg/L) were lower in the water of subjects who reported hypertension than those who did not (As: (*n* = 572, *M* = 0.008 mg/L, SD = 0.016 mg/L; *t*(602) = 2.82, **p** **=** **0.007**); Mn: (*n* = 572, *M* = 0.169 mg/L, SD = 0.269 mg/L; *t*(602) = 4.08, **p** **<** **0.001**); U (*n* = 572 = 4.84, *M* = 0.0013 mg/L, SD = 0.0017 mg/L; *t*(602) = 4.84, **p** **<** **0.001**). The concentrations of antimony, boron, barium, copper, lead, iron, vanadium, and zinc did not differ significantly in the water of subjects who reported hypertension and those who did not (data not shown).

Subjects who reported hypertension were older on average (*n* = 31, *M* = 51.7 years, SD = 10.4 years) than those who did not report hypertension (*n* = 572, *M* = 29.8, SD = 17.0; *t*(602) = −10.81, **p** **<** **0.001**). Subjects who reported hypertension were more likely to also report using tobacco than those who did not report hypertension (*χ*^2^(1,602) = 10.76, **p** **=** **0.001**). There was no difference in reporting hypertension between nonsmoking subjects living in households that contained at least one tobacco smoker and those that did not (*χ*^2^(1,541) = 0.0004, *p* = 0.985). The prevalence of subjects reporting hypertension did not differ by gender (*χ*^2^(1,591) = 0.09, *p* = 0.763). The proportion of subjects who reported hypertension from Higher SES households was not different from that of Lower SES households (*χ*^2^(1,602) = 0.31, *p* = 0.577). An examination of mixed effects logistic regression models found a significant effect of age on hypertension after adjusting for household membership; see Table S3.

In Eastern Nepal, “High” SES was associated with greater chance of hypertension being diagnosed rather than unknown [118]. Increased risk of hypertension has been associated with older age, male gender, and tobacco use in other South Asian studies [117, 119, 120], although Chataut et al. found that tobacco use was not a significant factor for predicting hypertension prevalence after adjusting for gender [119]. In Bangladesh, blood pressure measurements were highly associated with sodium concentrations in drinking water [121]; we did not measure sodium concentrations in water samples, so this possible association with hypertension could not be tested.

#### 3.8.3. Common Cold

Common cold had a prevalence of 17% across all household members (101/603). This was within the ranges reported by other studies in Kathmandu Valley focusing on upper respiratory tract infections (URI) in children under 5 years old: 4.5% [122] to 23% [123]. Reported common cold prevalence was 17% (12/70) for those who used dug wells, 22% (35/157) for those who used stone spouts, and 14% (54/376) for those who used tube wells; the differences were not significant (*χ*^2^(1,602) = 5.01, *p* = 0.082). There was no difference in the number of households served by sources used by subjects who reported colds and those who did not (*t*(602) = −2.18, *p* = 0.031), after correcting for FDR. There was no difference in the temperature of the water at sources used by subjects who reported colds and those who did not report colds (*t*(602) = −0.33, *p* = 0.741). Total coliform counts in a subject's water source did not differ between subjects who reported colds and those who did not (*t*(602) = 0.22, *p* = 0.822), but fecal coliform counts were lower in the water of subjects who reported colds (*n* = 101, *M* = 175.1, SD = 551.7) than those who did not (*n* = 502, *M* = 381.3, SD = 892.6; *t*(602) = 3.04, **p** **=** **0.003**). The concentration of Mn (*n* = 101, *M* = 0.266 mg/L, SD = 0.434 mg/L) was lower in the water used by subjects reporting colds than those who did not (Mn: *n* = 502, *M* = 0.414 mg/L, SD = 0.755 mg/L; *t*(602) = 2.71, **p** **=** **0.007**). The concentrations of As, B, Ba, Cu, Fe, Pb, Sb, V, U, and Zn did not differ in the water of subjects who reported colds and those who did not (data not shown).

Subjects who reported colds were younger on average (*n* = 101, *M* = 26.8 years, SD = 15.7 years) than those who did not (*n* = 502, *M* = 31.8 years, SD = 17.6 years; *t*(602) = 2.84, **p** **=** **0.005**). The prevalence of colds was no different between subjects who used tobacco and those who did not (*χ*^2^(1,602) = 0.07, *p* = 0.795), or between nonsmoking subjects who had the potential for secondhand smoke in their households and those who did not (*χ*^2^(1,541) = 0.65, *p* = 0.421). Subjects who reported colds lived in households where the most educated person in the household had fewer years of schooling (*n* = 101, *M* = 11.28 years, SD = 4.4 years) than those who did not report colds (*n* = 502, *M* = 12.7 years, SD = 4.1 years; *t*(602) = 2.96, **p** **=** **0.004**), but among subjects ages 15 and older who reported colds and those who did not, there was no difference in number of years of individual schooling (*t*(492) = 1.36, *p* = 0.175). Subjects who reported colds came from smaller households (*n* = 101, *M* = 4.1 people, SD = 1.3) than those who did not report colds (*n* = 502, *M* = 5.1 people, SD = 1.9; *t*(602) = 6.33, **p** **<** **0.001**). The ratio of the subjects who reported colds from Higher SES households was not different from that of Lower SES households (*χ*^2^(1,602) = 1.03, *p* = 0.310).

An examination of mixed effects logistic regression models found significant effects of household size and maximum years of schooling in the household on prevalence of colds after adjusting for household membership; see Table S4. Other studies in Kathmandu Valley have found associations between respiratory infection prevalence, younger age, lower SES, and air quality [122–125]. In contrast to our study, which included adults, in a study of children under 5 years old in Kathmandu Valley, larger households were associated with greater prevalence of respiratory infections [125].

#### 3.8.4. Schooling Attitude

Of the 443 individuals who had ever attended school and responded to our survey question about schooling attitude, 405 reported having a positive attitude towards school, 35 a neutral attitude, and 3 a negative attitude; that is, 91% had a positive attitude towards school, and 9% had a nonpositive attitude. A positive schooling attitude was reported for 95% of subjects who used dug wells, 86% of subjects who used stone spouts, and 93% of subjects who used tube wells; the differences were not significant (*χ*^2^(2,442) = 5.48, *p* = 0.065). Total coliform counts in a subject's water source did not differ between subjects who were reported as having a positive attitude towards school and those who did not (*t*(442) = −0.90, *p* = 0.375), but fecal coliform counts were higher in the water of subjects who reported a positive schooling attitude (*n* = 405, *M* = 362.2, SD = 893.9) than those who did not (*n* = 38, *M* = 175.0, SD = 399.8; *t*(442) = −2.38, **p** **=** **0.020**). The concentrations of As (*n* = 405, *M* = 0.009 mg/L, SD = 0.017 mg/L), B (*n* = 405, *M* = 0.056 mg/L, SD = 0.041 mg/L), Fe (*n* = 405, *M* = 1.57 mg/L, SD = 2.2 mg/L), and Mn (*n* = 405, *M* = 0.436 mg/L, SD = 0.763 mg/L) were higher, while U (*n* = 38, *M* = 0.001 mg/L, SD = 0.002 mg/L) was lower in the water used by subjects who reported a positive attitude towards school compared to the water of subjects who did not (As: *n* = 38, *M* = 0.002 mg/L, SD = 0.001 mg/L; *t*(442) = −8.51, **p** **<** **0.001**; B: *n* = 38, *M* = 0.041 mg/L, SD = 0.034 mg/L; *t*(442) = −2.62, **p** **=** **0.012**; Fe: *n* = 38, *M* = 0.8 mg/L, SD = 1.6 mg/L; *t*(442) = −2.84, **p** **=** **0.006**; Mn: *n* = 38, *M* = 0.125 mg/L, SD = 0.138 mg/L; *t*(442) = −7.06, **p** **<** **0.001**; U: *n* = 38, *M* = 0.002 mg/L, SD = 0.002 mg/L; *t*(442) = 2.77, **p** **=** **0.008**). The concentrations of Ba, Cu, Pb, Sb, V, and Zn did not significantly differ in the water of subjects who had a positive attitude towards school and those who did not (data not shown).

Subjects who had a positive attitude towards school were older (*n* = 405, *M* = 28.6 years, SD = 16.0 years) than those who did not (*n* = 38, *M* = 22.8 years, SD = 10.5 years; *t*(442) = −3.12, **p** **=** **0.003**). There was no gender difference among subjects having a positive attitude towards school (*χ*^2^(394) = 0.92, *p* = 0.337). Subjects who had a positive attitude towards school were no less likely to use tobacco than those who did not have a positive attitude (*χ*^2^(1,404) = 0.065, *p* = 0.798). Subjects who had a positive schooling attitude had completed more years of schooling (*n* = 405, *M* = 10.6 years, SD = 4.4 years) than those who did not (*n* = 38, *M* = 8.1 years, SD = 3.5 years; *t*(442) = −4.09, **p** **<** **0.001**), and they lived in households whose most educated member had completed more years of school (*n* = 405, *M* = 13.6 years, SD = 3.4 years) than those who did not (*n* = 38, *M* = 10.5 years, SD = 4.9 years; *t*(442) = −6.00, **p** **<** **0.001**). Subjects who had a positive schooling attitude came from households with more members (*n* = 405, *M* = 5.0, SD = 1.9) than those who did not (*n* = 38, *M* = 4.3, SD = 1.3; *t*(442) = −3.08, **p** **=** **0.003**). There was no difference in SES (Higher, Lower) among subjects who had a positive attitude towards school (*χ*^2^(1,442) = 0.40, *p* = 0.526).

An examination of mixed effects logistic regression models found significant effects of maximum years of schooling in the household and the square of drinking water Mn concentration on having a positive attitude towards school after adjusting for household membership; see Table S5. This finding is consistent with prior research that has found U-shaped relationships between Mn biomarkers and neurodevelopmental outcomes [126, 127]. Numerous studies have reported associations between excess Mn exposure in childhood and neurodevelopmental problems including Intelligence Quotient (IQ) deficits, impaired executive function, and ADHD [36, 37, 127].

### 3.9. Limitations of This Study

This study had several limitations, which may restrict how well the results can be generalized beyond this population. Due to financial constraints, only 35 water samples could be analyzed for metal contaminants, which may have limited the statistical power to detect differences in associations between water source types and water contaminants. We did not measure TDS or sodium or do speciation studies, which may have limited our ability to discover associations between the chemical composition of the water and taste ratings. We also did not ask consumers for visual assessment of the water quality.

The devastation to infrastructure due to the April 2015 earthquake impeded access to some neighborhoods and their water sources. This may be one reason why our sample of households included a greater proportion of Higher SES households than we had expected according to contemporary statistical surveys of the Kathmandu Valley [56, 91]. The SES of our sample of households was higher than the general population, so our findings may be more relevant for higher SES households. Since drinking water represents only one source of exposure to the contaminants that we measured, and the health effects that we studied may have multiple causes, associations between specific contaminants and specific health effects may have been too weak to detect within our sample of 603 individuals. We also relied on self-reporting of health conditions. We have no basis to evaluate the accuracy of these self-reports; in the case of hypertension, it is highly likely that self-reports of hypertension greatly underestimated actual prevalence.

## 4. Conclusions

Most water used for drinking in the Kathmandu Valley contains bacterial and/or chemical contaminants that make it unsafe for human health. Some water sources in the Kathmandu Valley contain unsafe concentrations of arsenic, manganese, and/or iron. High concentrations of these metals are often found in water from sources located in deltaic and floodplain sediments, the concentrations tend to increase with depth of the water source, and the metals are likely released under reducing conditions.

In this study, we found both high and low Mn concentrations in water sources associated with a less positive attitude towards schooling, consistent with prior reports of biphasic or U-shaped relationships between Mn biomarkers and neurodevelopmental measures [126, 127]. We did not find significant effects of other water contaminants on the health conditions studied (GI symptoms, hypertension, and common colds) after adjusting for water source, household, and social factors. We found that education at the individual or household level is a key factor for health, associated with significantly lower common cold and GI symptom prevalence and tobacco usage.

In this study, we did not find evidence for water contaminants (coliform or chemical) affecting water taste. In particular, taste ratings were not affected by iron or manganese concentrations, and consumers did not consistently rate water with unsafe concentrations of iron or manganese (exceeding WHO HBVs) as having poor taste. While taste is a stated factor in water source decisions for many users, the cost of water may outweigh taste for the most disadvantaged users, as suggested by our finding that Lower SES households were more likely to mention cost of water when explaining their water source preferences. In addition, many of the samples came from public drinking water sources where the aesthetic concerns of potential staining of plumbing fixtures or laundry from high concentrations of iron or manganese are not applicable. Thus, the WHO's current reliance on acceptability concerns to deter consumers from drinking water with concentrations of iron and manganese high enough to cause health risks may not be sufficient to protect public health [17, 81]. Relying on acceptability concerns to deter consumers from unsafe water may be particularly hazardous for the most-economically disadvantaged, since they may be more willing to drink water with marginal taste.

## Figures and Tables

**Table 1:** Sample sources, characteristics, bacterial counts, and taste ratings.

Sample	Latitude	Longitude	Well type	Depth (1)	Ownership	Years in service	House-holds served	Total coliform (CFU/100 mL)	Fecal coliform (CFU/100 mL)	Taste rating average (2)
W01	27°43′15.96″N	85°18′9.1°8″E	Tube well	Shallow	Private	10	5	280	0	2.2
W02	27°43′35.04″N	85°18′16.41″E	Tube well	Shallow	Public	50	30	10	290	2
W03	27°43′17.76″N	85°18′17.964″E	Tube well	Shallow	Private	9	8	320	30	2.6
W04	27°42′59.112″N	85°18′12.492″E	Tube well	Shallow	Private	5	7	210	370	1.6
W05	27°43′13.35″N	85°18′5.5″E	Tube well	Shallow	Public	30	40	1,700	10	2.4
W06	27°42′12.10″N	85°18′19.12″E	Tube well	Shallow	Public	100	90	120	100	2.2
W07	27°42′9.36″N	85°18′19.08″E	Stone spout	Surface	Public	300	175	90	10	1.8
W08	27°42′16.16″N	85°18′19.08″E	Dug well	Surface	Public	100	100	1,700	100	2
W09	27°41′54.10″N	85°18′00.27″E	Dug well	Surface	Private	18	1	300	320	2
W10	27°42′26.07″N	85°18′26.98″E	Tube well	Shallow	Public	100	60	1,110	0	2.4
W11	27°42′30.49″N	85°18′45.83″E	Tube well	Shallow	Public	100	60	200	0	2.6
W12	27°42′30.69″N	85°21′12.27″E	Tube well	Deep	Public	6	500	1	0	2.8
W13	27°42′32.82″N	85°18′43.20″E	Tube well	Shallow	Public	28	40	180	0	2.8
W14	27°42′37.22″N	85°18′37.30″E	Tube well	Shallow	Public	100	55	1,700	3,025	2.8
W15	27°41′15.59″N	85°19′17.46″E	Tube well	Shallow	Private	30	15	20	0	2.4
W16	27°41′16.66″N	85°19′20.46″E	Tube well	Shallow	Private	20	6	380	40	1.8
W17	27°41′13.78″N	85°20′3.19″E	Stone spout	Surface	Public	100	150	320	1,310	2.6
W18	27°41′44.88″N	85°20′15.88″E	Stone spout	Surface	Public	300	90	1,700	30	2.6
W19	27°42′22.30″N	85°20′50.88″E	Tube well	Deep	Public	7	5	120	51	2
W20	27°40′12.39″N	85°21′4.13″E	Tube well	Shallow	Private	30	6	67	3,025	1.6
W21	27°42′48.40″N	85°19′11.12″E	Stone spout	Surface	Public	120	100	32	72	1.8
W22	27°42′50.72″N	85°19′34.35″E	Tube well	Shallow	Public	6	100	57	45	2
W23	27°43′13.50″N	85°19′51.93″E	Stone spout	Surface	Public	150	350	5	4	2.2
W24	27°43′15.47″N	85°20′7.90″E	Dug well	Surface	Private	16	7	75	54	2
W25	27°43′38.47″N	85°20′12.45″E	Stone spout	Surface	Public	100	125	1,700	3	2.4
W26	27°43′44.99″N	85°20′16.89″E	Stone spout	Surface	Public	10	200	1,700	0	2.2
W27	27°44′13.90″N	85°19′38.78″E	Tube well	Shallow	Private	12	7	1,700	1	1
W28	27°42′18.86″N	85°19′32.28″E	Dug well	Surface	Private	100	6	53	19	2.2
W29	27°41′34.39″N	85°19′52.61″E	Stone spout	Surface	Public	50	100	1,700	14	2.4
W30	27°42′18.95″N	85°19′35″E	Tube well	Deep	Private	13	15	0	0	2.2
W31	27°42′14.54″N	85°18′12.64″E	Dug well	Surface	Private	15	3	560	110	3
W32	27°42′9.36″N	85°18′15.66″E	Stone spout	Surface	Public	200	90	1,680	3,020	3
W33	27°42′0.36″N	85°18′22.72″E	Dug well	Shallow	Public	100	55	1,000	980	2
W34	27°41′56.36″N	85°18′01.36″E	Dug well	Shallow	Public	48	7.5	240	190	1
W35	27°41′16.66″N	85°19′20.46″E	Tap water	Unknown	Public	10	8	0	0	2.9
					**Mean**	68.4	74.8	600.9	377.8	2.2
					**Min**	5	1	0	0	1
					**Max**	300	500	1,700	3,025	3

(1) “Deep” was defined as 8 meters or more. Stone spouts were classified as “surface” sources, but the depth of these sources was unknown; only depths of dug wells and tube wells were used for statistical analyses. (2) For this table only, “poor” was assigned 1, “OK” 2, “Good” 3. These numerical taste scores were averaged across all responding households for each source only to provide concise summary results for this ordinal variable. Our formal statistical tests treated taste ratings as categorical variables rather than as numerical scores.

**Table 2:** Chemical contaminants, drinking water standards, health-based values, and guidelines.

Contaminant	Min (mg/L)	Max (mg/L)	Mean (mg/L)	Nepal DWQS (mg/L) (1)	Samples exceeding Nepal DWQS (%)	WHO HBV (mg/L) (2)	Samples exceeding WHO HBV (%)	WHO DWG (mg/L) (2)	Samples exceeding WHO DWG (%)
Aluminum	<0.02	0.53	0.05	0.2	3 (9%)	0.9	0 (0%)	na	na
Arsenic	<0.001	0.071	0.007	0.05	1 (3%)	nd (3)	na	0.01	3 (9%)
Antimony	<0.001	0.002	<0.001	na	na	0.02	0 (0%)	0.02	0 (0%)
Barium	<0.01	0.44	0.08	na	na	1.3	0 (0%)	1.3	0 (0%)
Beryllium	<0.001	<0.001	<0.001	na	na	0.012	0 (0%)	na	na
Boron	0.008	0.154	0.055	na	na	2.0 (4)	0 (0%)	2.4	0 (0%)
Cadmium	<0.001	<0.001	<0.001	0.003	0 (0%)	0.003	0 (0%)	0.003	0 (0%)
Chromium	<0.01	<0.01	<0.01	0.05	0 (0%)	0.05	0 (0%)	0.05	0 (0%)
Cobalt	<0.01	<0.01	<0.01	na	na	na	na	na	na
Copper	<0.02	0.05	<0.02	na	na	2	0 (0%)	2	0 (0%)
Fluoride	<0.3	<0.3	<0.3	1.5	0 (0%)	1.5	0 (0%)	1.5	0 (0%)
Iron	<0.10	7.22	1.21	0.3	16 (46%)	2	7 (20%)	na	na
Lead	<0.001	0.003	<0.001	0.01	0 (0%)	<0.01	0 (0%)	0.01	0 (0%)
Manganese	<0.005	3.229	0.350	0.2	12 (34%)	0.4	7 (20%)	na	na
Mercury (total Hg)	<0.0005	<0.0005	<0.0005	0.001 (total Hg)	0 (0%)	0.006 (inorganic Hg)	0 (0%)	0.006 (inorganic Hg)	0 (0%)
Molybdenum	<0.005	0.031	0.001	na	na	0.07	0 (0%)	na	0 (0%)
Nickel	<0.01	0.01	<0.01	na	na	0.07	0 (0%)	0.07	0 (0%)
Selenium	<0.005	<0.005	<0.005	na	na	0.04	0 (0%)	0.04	0 (0%)
Uranium	<0.001	0.007	0.001	na	na	0.03	0 (0%)	0.03	0 (0%)
Thallium	<0.001	<0.001	<0.001	na	na	na	na	na	na
Vanadium	<0.005	0.025	0.005	na	na	na	na	na	na
Zinc	<0.02	0.45	0.02	3	0 (0%)	3	0 (0%)	na	na

DWQS: drinking water quality standards; WHO: World Health Organization; HBV: health-based value; DWG: drinking-water guideline; na = not applicable; nd = not determined; ≤less than the limit of detection. (1) Government of Nepal [80]. (2) WHO [17]; WHO [81]. (3) WHO [82]. (4) WHO [17]; WHO [81]; Frisbie et al. [83].

## Data Availability

The data are available from the corresponding author upon request.

## Abbreviations

AAS: Atomic absorption spectrophotometer
ADHD: Attention deficit hyperactivity disorder
As: Arsenic
Au: Gold
B: Boron
Ba: Barium
Be: Beryllium
Ca: Calcium
Cd: Cadmium
CFU: Colony forming unit
Co: Cobalt
Cr: Chromium
Cu: Copper
DWG: Drinking-water guidelines
DWQS: Nepal drinking water quality standards
ENPHO: Environment and public health organization
ESI: Elemental Scientific Inc.
F^−^: Fluoride anion
FAAS: Flame atomic absorption spectrometry
FDR: False detection rate
Fe: Iron
FIA: Flow injection analyzer
GI: Gastro-intestinal
GPS: Global positioning system
HBV: Health-based values
Hg: Mercury
HNO_3_: Nitric acid
ICP/MS: Inductively coupled plasma/mass spectrometry
IQ: Intelligence quotient
Li: Lithium
m: Meters
*M*: Sample mean
Mg: Magnesium
Mg: Magnesium
mg/L: Milligrams per liter
mL: Milliliter
Mn: Manganese
Mo: Molybdenum
*N*: Sample size
*n*: Sample size of subgroup
Na: Sodium
NELAP: National Environmental Laboratory Accreditation Program
Ni: Nickel
NO_3_^−^: Nitrate
OR: Odds ratio
*p*: *p* value
Pb: Lead
QA/QC: Quality assurance/quality control
Sb: Antimony
SD: Sample standard deviation
SDWA: Safe drinking water act
Se: Selenium
SES: Socioeconomic status
*t*: *t*-score
TDS: Total dissolved solids
Tl: Thallium
U.S. EPA: United States environmental protection agency
U: Uranium
URI: Upper respiratory tract infections
USGS: United States geological survey
V: Vanadium
VDHL: Vermont department of health laboratory
VT: Vermont
WHO: World Health Organization
Zn: Zinc
*χ* ^2^: Chi squared
*μ*g/L: Micrograms per liter.

## Supplementary Materials

Supplementary Materials Table S1: Population characteristics. Table S2: Mixed effects multiple logistic regression analysis for reported use of tobacco among Kathmandu Valley subjects 12 years and older. Table S3: Mixed effects multiple logistic regression analysis for reported hypertension among Kathmandu Valley subjects. Table S4: Mixed effects multiple logistic regression analysis for reported prevalence of colds among Kathmandu Valley subjects. Table S5: Mixed effects multiple logistic regression analysis for reported positive attitude towards school among Kathmandu Valley subjects who have ever attended school.
