# Supplementary material for: Drinking Water Quality and Public Health in the Kathmandu Valley, Nepal: Coliform Bacteria, Chemical Contaminants, and Health Status of Consumers
Source: J Environ Public Health. 2022 Feb 12;2022:3895859. doi: 10.1155/2022/3895859 (PMC8858048; doi:10.1155/2022/3895859)
Supplement: Supplementary Materials — Table S1: Population characteristics. Table S2: Mixed effects multiple logistic regression analysis for reported use of tobacco among Kathmandu Valley subjects 12 years and older. Table S3: Mixed effects multiple logistic regression analysis for reported hypertension among Kathmandu Valley subjects. Table S4: Mixed effects multiple logistic regression analysis for reported prevalence of colds among Kathmandu Valley subjects. Table S5: Mixed effects multiple logistic regression analysis for reported positive attitude towards school among Kathmandu Valley subjects who have ever attended school. [file 3895859.f1.zip › 3895859.f1/table s1 may 2021 (3).docx]

Table S1: Population Characteristics

|  |  | **Total Population** | **GI symptoms within last 4 weeks** | **High blood pressure** | **Common cold** | **Use tobacco** | **Ever attended school** | **Mean years attended school** | **Negative schooling attitude** | **Neutral schooling attitude** | **Positive schooling attitude** |
| --- | --- | --- | --- | --- | --- | --- | --- | --- | --- | --- | --- |
| All subjects |  | 603 | 105 | 31 | 101 | 61 | 456 | 8 | 3 | 37 | 416 |
| By Age |  |  |  |  |  |  |  |  |  |  |  |
|  | Ages 0-5 years | 28 | 6 | 0 | 10 | 0 | 18 | 0.4 | 0 | 2 | 16 |
|  | Ages 6-14 years | 38 | 15 | 0 | 16 | 1 | 78 | 5.1 | 1 | 5 | 72 |
|  | Ages 15 years + | 537 | 84 | 31 | 75 | 60 | 360 | 8.8 | 2 | 30 | 328 |
| By SES |  |  |  |  |  |  |  |  |  |  |  |
|  | Higher SES | 451 | 70 | 25 | 71 | 38 | 362 | 8.4 | 1 | 29 | 332 |
|  | Lower SES | 152 | 35 | 6 | 30 | 23 | 94 | 6.6 | 2 | 8 | 84 |
| By Gender |  |  |  |  |  |  |  |  |  |  |  |
|  | Female | 292 | 57 | 14 | 51 | 8 | 207 | 7.2 | 2 | 20 | 185 |
|  | Male | 299 | 48 | 17 | 48 | 53 | 238 | 8.6 | 1 | 17 | 220 |
|  | (gender was not specified for some individuals) | | | |  |  |  |  |  |  |  |
