# Supplementary material for: Drinking Water Quality and Public Health in the Kathmandu Valley, Nepal: Coliform Bacteria, Chemical Contaminants, and Health Status of Consumers
Source: J Environ Public Health. 2022 Feb 12;2022:3895859. doi: 10.1155/2022/3895859 (PMC8858048; doi:10.1155/2022/3895859)
Supplement: Supplementary Materials — Table S1: Population characteristics. Table S2: Mixed effects multiple logistic regression analysis for reported use of tobacco among Kathmandu Valley subjects 12 years and older. Table S3: Mixed effects multiple logistic regression analysis for reported hypertension among Kathmandu Valley subjects. Table S4: Mixed effects multiple logistic regression analysis for reported prevalence of colds among Kathmandu Valley subjects. Table S5: Mixed effects multiple logistic regression analysis for reported positive attitude towards school among Kathmandu Valley subjects who have ever attended school. [file 3895859.f1.zip › 3895859.f1/table s2 may 2021 (2).docx]

Table S2. Mixed effects multiple logistic regression analysis for reported use of tobacco among Kathmandu Valley subjects 12 years and older

| Variable |  | Odds Ratio | 95% Confidence Interval | *p*-value |
| --- | --- | --- | --- | --- |
| Male gender |  | 10.16 | 4.23 to 24.40 | <0.01 |
| Age |  | 1.05 | 1.03 to 1.08 | <0.01 |
|  |  |  |  |  |
| Household membership (random effect, n=145) |  |  |  |  |
| AIC | 310.69 |  |  |  |
| BIC | 326.68 |  |  |  |
| Pseudo R^2^ (fixed effects) | 0.30 |  |  |  |
| Pseudo R^2^ (total) | 0.54 |  |  |  |
| n | 516 |  |  |  |

2
