# Supplementary material for: Drinking Water Quality and Public Health in the Kathmandu Valley, Nepal: Coliform Bacteria, Chemical Contaminants, and Health Status of Consumers
Source: J Environ Public Health. 2022 Feb 12;2022:3895859. doi: 10.1155/2022/3895859 (PMC8858048; doi:10.1155/2022/3895859)
Supplement: Supplementary Materials — Table S1: Population characteristics. Table S2: Mixed effects multiple logistic regression analysis for reported use of tobacco among Kathmandu Valley subjects 12 years and older. Table S3: Mixed effects multiple logistic regression analysis for reported hypertension among Kathmandu Valley subjects. Table S4: Mixed effects multiple logistic regression analysis for reported prevalence of colds among Kathmandu Valley subjects. Table S5: Mixed effects multiple logistic regression analysis for reported positive attitude towards school among Kathmandu Valley subjects who have ever attended school. [file 3895859.f1.zip › 3895859.f1/table s4 may 2021 (2).docx]

Table S4. Mixed effects multiple logistic regression analysis for reported prevalence of colds among Kathmandu Valley subjects

| Variable |  | Odds Ratio | 95% Confidence Interval | *p*-value |
| --- | --- | --- | --- | --- |
| Household size |  | 0.70 | 0.59 to 0.82 | <0.01 |
| Age |  | 0.98 | 0.97 to 1.00 | 0.02 |
|  |  |  |  |  |
| Household membership (random effect, n=145) |  |  |  |  |
| AIC | 517.36 |  |  |  |
| BIC | 534.96 |  |  |  |
| Pseudo R^2^ (fixed effects) | 0.14 |  |  |  |
| Pseudo R^2^ (total) | 0.22 |  |  |  |
| n | 601 |  |  |  |
